# Practice patterns and outcomes of chemoradiotherapy versus radiotherapy alone for older patients with nasopharyngeal cancer

**DOI:** 10.1002/cam4.1290

**Published:** 2018-03-30

**Authors:** Vivek Verma, Swati M. Surkar, Amy C. Moreno, Chi Lin, Charles B. Simone

**Affiliations:** ^1^ Department of Radiation Oncology University of Nebraska Medical Center Omaha Nebraska; ^2^ Department of Physical Therapy Washington University School of Medicine St. Louis Missouri; ^3^ Department of Radiation Oncology University of Texas MD Anderson Cancer Center Houston Texas; ^4^ Department of Radiation Oncology University of Maryland Medical Center Baltimore Maryland

**Keywords:** Chemotherapy, elderly, geriatric, nasopharyngeal cancer, nasopharynx, radiation therapy

## Abstract

Older patients are at increased risk of toxicities from aggressive oncologic therapy and of nononcologic death. A meta‐analysis of non‐nasopharyngeal head and neck cancers showed no statistical benefit in adding chemotherapy to radiotherapy (RT) in older patients; another meta‐analysis of RT versus chemoradiotherapy (CRT) in NPC found advantages to CRT, but vastly under‐represented patients ≥70 years old. This is the largest study to date evaluating outcomes of CRT versus RT alone in this population. The National Cancer Data Base (NCDB) was queried for primary nasopharyngeal cancer cases (2004–2013) in patients ≥70 years old receiving RT alone or CRT. Patients with unknown RT/chemotherapy and T1N0 or M1 disease were excluded. Logistic regression analysis ascertained factors associated with CRT delivery. Kaplan–Meier analysis evaluated overall survival (OS) between both cohorts. Cox proportional hazards modeling determined variables associated with OS. In total, 930 patients were analyzed (*n* = 713 (77%) CRT,* n* = 217 (23%) RT). Groups were relatively balanced; CRT was less frequently delivered in patients with advancing age, lower nodal burden, and females (*P* < 0.05 for all). Median OS in the CRT and RT groups were 35.3 versus 20.0 months, respectively (*P* = 0.002). On multivariate analysis, independent predictors of OS included age, comorbidities, income and insurance status, tumor grade, and stage (*P* < 0.05 for all). Notably, receipt of chemotherapy independently predicted for improved OS (*P* = 0.036). CRT, compared to RT alone, was independently associated with improved survival in NPC patients ≥70 years old. CRT appears to be a promising approach in this population, but treatment‐related toxicity risks should continue to be weighed against potential oncologic benefits.

## Introduction

Numerous clinical trials have demonstrated survival improvements with the addition of chemotherapy to definitive radiation therapy (RT) for head and neck cancers, as highlighted by the MACH‐NC meta‐analysis 1. One important result of this study, however, was a decreasing benefit to chemotherapy with increasing age, which was independent of other covariates analyzed. Moreover, nearly 40% of deaths in patients ≥71 years of age therein were not cancer‐related. Although the meta‐analysis did not evaluate toxicities, numerous studies have illustrated decreased tolerance of oncologic therapies by older patients 2, 3, 4.

A factor limiting applicability of the MACH‐NC report was its specific exclusion of nasopharyngeal cancer (NPC). This neoplasm, rare in the United States but endemic in south China and north Africa, is most commonly treated with chemoradiotherapy (CRT) 5. This paradigm is supported by the MAC‐NPC meta‐analysis, which demonstrated an overall survival (OS) benefit of 6% at 5 years with the addition of chemotherapy to RT 6. However, that analysis notably underrepresented older patients, likely related to the median age of NPC patients being approximately 55 years (in the United States) 7. Just 13% of the total MAC‐NPC cohort was ≥60 years of age; although the proportion ≥70 years old was not reported, it is likely less than half of that figure.

As a result, optimal management for older (defined as ≥70 years old herein) NPC patients with respect to the additional chemotherapy is currently not well defined. Although challenging to assess with single‐ or multi‐institutional analyses owing to the relative rarity of older NPC patients, the National Cancer Data Base (NCDB) provides a unique resource with which to address this novel but clinically important issue. In this investigation, the largest such study to date, we evaluated national practice patterns and outcomes in older NPC patients treated with CRT versus RT alone.

## Materials & Methods

The NCDB is a joint project of the Commission on Cancer (CoC) of the American College of Surgeons and the American Cancer Society, which consists of de‐identified information regarding tumor characteristics, patient demographics, and patient survival for approximately 70% of the United States population 8, 9, 10, 11, 12, 13, 14, 15. All pertinent cases are reported regularly from CoC‐accredited centers and compiled into a unified dataset, which is then validated. The NCDB contains information not included in the Surveillance, Epidemiology, and End Results database, including details regarding use of systemic therapy. The data used in the study were derived from a de‐identified NCDB file (2004–2013). The American College of Surgeons and the CoC have not verified and are neither responsible for the analytic or statistical methodology employed nor the conclusions drawn from these data by the investigators. As all patient information in the NCDB database is de‐identified, this study was exempt from institutional review board evaluation.

Inclusion criteria for this study were patients ≥70 years of age with newly‐diagnosed nasopharyngeal cancer treated with RT for curative intent. The 70‐year‐old threshold was utilized because it is among the most commonly used cutoff to denote “older” patients in head/neck cancer as well as many other areas of oncology 16; it is also close to the threshold utilized in the MACH‐NC study 1. T1N0 and M1 cases were excluded because CRT is not the consensus‐based recognized treatment for these subsets; patients receiving any form of pharyngectomy were similarly removed because it is nonstandard for NPC and to isolate the effect of adding chemotherapy to RT 5. Other exclusion criteria were incomplete staging information, palliative care treatment, and unknown information on RT and/or chemotherapy. In accordance with the variables in NCDB files, information collected on each patient broadly included demographic, clinical, and treatment data.

All statistical tests were performed with SPSS software (IBM Corporation, Armonk, NY); tests were two‐sided, with a threshold of *P* < 0.05 for statistical significance. Univariable and multivariable logistic regression were used to determine characteristics associated with receipt of CRT. All initially examined variables were considered for inclusion into models for stepwise selection. Survival analysis (performed using Kaplan–Meier methodology) evaluated OS, defined as the interval between the date of diagnosis and the date of death or censored at last contact. Univariate and multivariate Cox proportional hazards modeling was utilized to evaluate predictors of OS.

## Results

**Figure 1:**
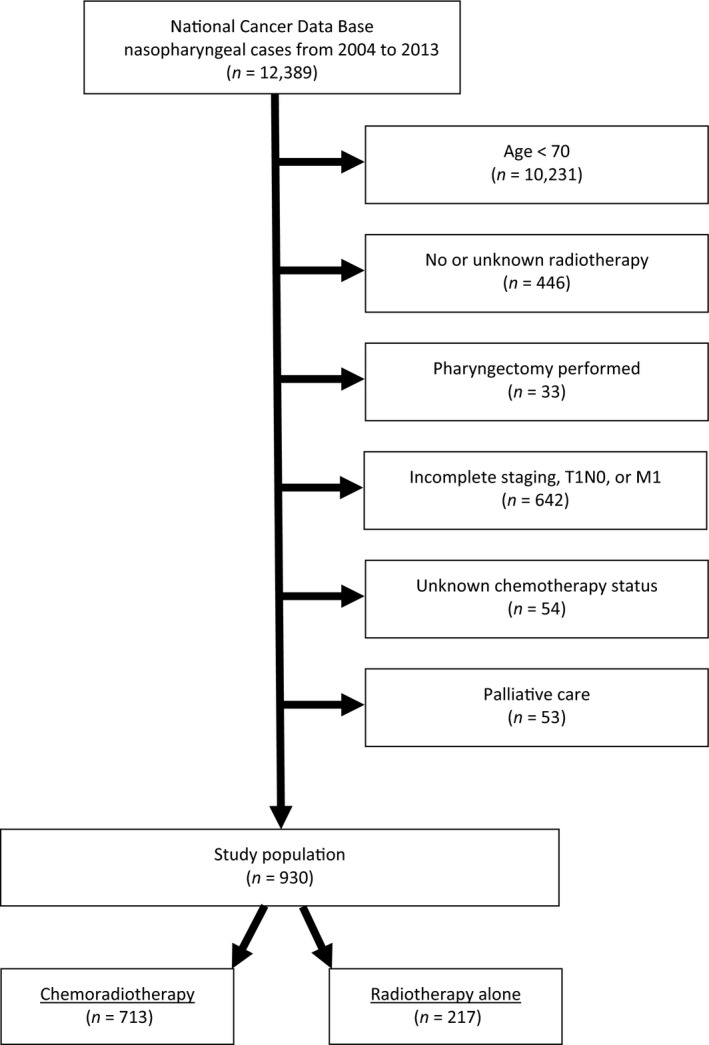
Patient selection diagram.

A complete flow diagram of patient selection is provided in Figure 1. In total, 930 patients met study analysis criteria. Table 1 displays notable clinical characteristics of the analyzed patients, most (74%) of whom were 70–79 years of age. A total of 713 (77%) patients underwent CRT, whereas 217 (23%) received RT alone. After univariable analysis was performed to assess factors associated with receipt of CRT, multivariable assessment revealed that factors independently associated with decreased likelihood of CRT delivery were advancing age (*P* = 0.001), female gender (*P* = 0.014), and node‐negative disease (*P* = 0.002). There was also a trend toward increasing CRT receipt in more recent years (2009–2013, *P* = 0.063).

**Table 1:** Characteristics of the overall cohort and factors associated with receiving chemoradiotherapy

Parameter, *N* (%) or median (range)	CRT(*N* = 713)	RT Alone(*N* = 217)	Univariable		Multivariable (stepwise)	
			OR (95% CI)	*P*‐value	OR (95% CI)	*P*‐value
Age (years)						
Median (range)	75 (70–90)	79 (70–90)	0.542 (0.398–0.737)	**0.001**	0.860 (0.832–0.888)	**0.001**
Gender						
Male	467 (66%)	110 (51%)	1.161 (1.125–1.198)	**0.001**	1.534 (1.089–2.161)	**0.014**
Female	246 (34%)	107 (49%)	REF		REF	
Race						
White	522 (73%)	166 (76%)	REF			
Black	65 (9%)	18 (8%)	0.871 (0.502–1.510)	0.622		
Other	106 (15%)	26 (12%)	0.771 (0.485–1.226)	0.272		
Unknown	20 (3%)	7 (3%)	–	–		
Charlson deyo score^a^						
0	541 (76%)	158 (73%)	REF			
1	129 (18%)	43 (20%)	1.141 (0.774–1.682)	0.504		
≥2	43 (6%)	16 (7%)	1.274 (0.699–2.323)	0.429		
Insurance type						
Uninsured	3 (0%)	0 (0%)	–	–		
Private	88 (12%)	29 (13%)	1.098 (0.699–1.727)	0.684		
Medicaid/Other Government (non‐Medicare)	31 (4%)	10 (5%)	1.075 (0.517–2.237)	0.846		
Medicare	580 (81%)	174 (80%)	REF			
Unknown	9 (1%)	4 (2%)	–	–		
Income (US dollars/year)						
<$30,000	127 (18%)	44 (20%)	REF			
$30,000–$34,999	157 (22%)	52 (24%)	0.956 (0.601–1.521)	0.849		
$35,000–$45,999	195 (27%)	57 (26%)	0.844 (0.537–1.326)	0.462		
≥$46,000	226 (32%)	57 (26%)	0.728 (0.464–1.141)	0.166		
Unknown	6 (1%)	7 (3%)	–	–		
Location						
Metro	596 (84%)	174 (80%)	REF			
Urban	85 (12%)	31 (14%)	1.249 (0.801–1.949)	0.327		
Rural	9 (1%)	2 (1%)	0.761 (0.163–3.556)	0.761		
Unknown	23 (3%)	10 (5%)	–	–		
Percentage of adults in zip code without high school diploma						
≥21%	137 (19%)	38 (18%)	REF			
13–20.9%	186 (26%)	61 (28%)	1.182 (0.745–1.875)	0.477		
7–12.9%	233 (33%)	68 (31%)	1.052 (0.671–1.649)	0.825		
<7%	149 (21%)	44 (20%)	1.065 (0.651–1.742)	0.803		
Unknown	8 (1%)	6 (3%)	–	–		
Facility type						
Community	385 (54%)	134 (62%)	REF			
Academic	328 (46%)	83 (38%)	1.375 (1.008–1.877)	**0.044**		
Facility location						
Northeast	174 (24%)	39 (18%)	REF			
South	223 (31%)	76 (35%)	0.752 (0.461–1.227)	0.254		
Midwest	175 (25%)	60 (28%)	1.144 (0.743–1.762)	0.541		
West	141 (20%)	42 (19%)	1.151 (0.732–1.810)	0.542		
Distance to treating facility (mi)						
Median (range)	8 (0–2456)	7 (0–1736)	1.000 (0.999–1.001)	0.510		
Year of Diagnosis						
2004–2008	330 (46%)	117 (54%)	0.736 (0.543–0.999)	**0.049**	0.723 (0.514–1.017)	0.063
2009–2013	383 (54%)	100 (46%)	REF		REF	
Tumor grade						
Well or moderate	138 (19%)	38 (18%)	REF			
Poorly, undifferentiated, anaplastic	384 (54%)	99 (46%)	1.068 (0.701–1.628)	0.759		
Unknown	191 (27%)	80 (37%)	–	–		
T classification						
X	21 (3%)	6 (3%)	REF			
1	147 (21%)	33 (15%)	1.577 (0.282–8.808)	0.604		
2	203 (28%)	72 (33%)	0.708 (0.443–1.132)	0.149		
3	137 (19%)	41 (19%)	1.119 (0.759–1.648)	0.571		
4	205 (29%)	65 (30%)	0.944 (0.604–1.475)	0.800		
N classification						
0	193 (27%)	103 (47%)	0.396 (0.238–0.554)	**0.001**	0.252 (0.107–0.603)	**0.002**
1	237 (33%)	53 (24%)	0.630 (0.127–1.133)	0.238	0.570 (0.236–1.379)	0.213
2	196 (27%)	43 (20%)	0.661 (0.161–1.161)	0.263	0.614 (0.251–1.503)	0.263
3	52 (7%)	7 (3%)	REF		REF	
Unknown	35 (5%)	11 (5%)	–	–	–	–
Group stage						
II	94 (13%)	34 (16%)	1.370 (0.858–2.186)	0.187		
III	233 (33%)	64 (29%)	1.040 (0.714–1.515)	0.837		
IV	284 (40%)	75 (35%)	REF			
Unknown	102 (14%)	44 (20%)	–	–	–	–

Statistically significant *P*‐values (*P* < 0.05) are in bold. Only values included in the final multivariable model are shown. CRT, chemoradiotherapy; RT, radiotherapy; OR, odds ratio; CI, confidence interval.

a The Charlson‐Deyo index is a weighted score of comorbidities as defined by several medical codes.

**Figure 2:**
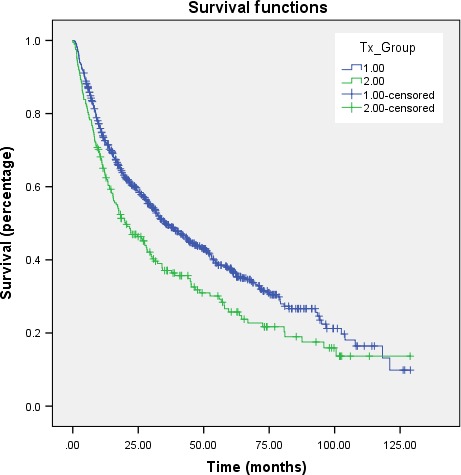
Kaplan–Meier overall survival curve comparing those receiving radiotherapy alone (green) versus chemoradiotherapy (blue).

Median follow‐up was 23 months (range, 0–129 months). Kaplan–Meier estimates comparing OS in patients that received RT alone versus CRT are illustrated in Figure 2; median OS in the respective cohorts were 20.0 (95% confidence interval (CI), 12.8–27.3) months and 35.3 (95% CI, 29.3–41.2) months (*P* = 0.002).

In the overall cohort, there were several predictors of OS on univariate analysis (Table 2). After multivariate adjustment for potential confounding factors (Table 2), factors independently associated with poorer OS included advancing age, comorbidity index, lower income, Medicare insurance (relative to private), poor/undifferentiated/anaplastic disease, and stage IV (M0) disease (*P* < 0.05 for all). Of note, receipt of CRT relative to RT alone independently predicted for improved OS (hazard ratio, 0.721, 95% CI, 0.532–0.979, *P* = 0.036).

**Table 2:** Univariate and multivariate Cox proportional hazards model for overall survival

Parameter	Univariate			Multivariate		
	HR	95% CI	*P*‐value	HR	95% CI	*P*‐value
Treatment group (CRT vs. RT alone)	0.735	0.606–0.892	**0.002**	0.721	0.532–0.979	**0.036**
Age (continuous)	1.053	1.036–1.069	**0.001**	1.063	1.037–1.090	**0.001**
Gender (male vs. female)	0.864	0.725–1.030	0.103			
Race (black vs. white)	0.845	0.625–1.144	0.276			
Race (others vs. white)	0.262	0.468–0.823	**0.001**			
Charlson–Deyo score (0 vs. 2)	0.517	0.377–0.710	**0.001**	0.517	0.377–0.710	**0.001**
Charlson–Deyo score (1 vs. 2)	0.609	0.426–0.872	**0.007**	0.609	0.426–0.872	**0.001**
Insurance (uninsured vs. Medicare)	2.252	0.560–9.052	0.253	2.752	0.374–20.234	0.320
Insurance (private vs. Medicare)	0.613	0.459–0.852	**0.001**	0.553	0.359–0.852	**0.007**
Insurance (Medicaid/other government vs. Medicare)	0.629	0.387–1.022	0.061	0.700	0.377–1.298	0.257
Income (<$30,000 vs. $30,000–$34,999)	1.584	1.233–2.035	**0.001**	1.584	1.233–2.035	**0.001**
Income (<$30,000 vs. $35,000–$45,999)	1.403	1.097–1.794	**0.019**	1.403	1.097–1.703	**0.007**
Income (<$30,000 vs. ≥$46,000)	1.323	1.047–1.671	**0.039**	1.323	1.047–1.671	**0.019**
Location (urban vs. metro)	1.384	1.080–1.773	**0.010**			
Location (rural vs. metro)	1.777	0.841–3.757	0.132			
Percentage of adults in zip code without high school diploma (13–20.9% vs. ≥21%)	1.125	0.851–1.486	0.408			
Percentage of adults in zip code without high school diploma (7–12.9% vs. ≥21%)	1.267	0.986–1.629	0.065			
Percentage of adults in zip code without high school diploma (<7% vs. ≥21%)	1.049	0.820–1.341	0.704			
Facility type (academic vs. community)	1.146	0.962–1.366	0.126			
Facility location (South vs. Northeast)	1.143	0.867–1.507	0.344			
Facility location (Midwest vs. Northeast)	1.289	1.001–1.658	**0.049**			
Facility location (West vs. Northeast)	1.301	1.000–1.693	**0.050**			
Distance to treatment facility (continuous)	1.000	0.999–1.000	0.515			
Year of diagnosis (2004–2008 vs. 2009–2013)	0.981	0.815–1.182	0.843			
Grade (poor/undifferentiated/anaplastic vs. well/moderate)	1.691	1.356–2.110	**0.001**	1.510	1.163–1.962	**0.002**
T classification (x vs. 1)	0.326	0.104–1.022	0.055			
T classification (x vs. 2)	0.404	0.311–0.525	**0.001**			
T classification (x vs. 3)	0.494	0.397–0.614	**0.001**			
T classification (x vs. 4)	0.532	0.411–0.698	**0.001**			
N classification (0 vs. 1)	0.548	0.379–0.792	**0.001**			
N classification (0 vs. 2)	0.695	0.481–1.003	0.052			
N classification (0 vs. 3)	0.612	0.426–0.879	**0.008**			
Group stage (II vs. IV)	0.497	0.358–0.691	**0.001**	0.530	0.359–0.783	**0.001**
Group stage (III vs. IV)	0.619	0.505–0.759	**0.001**	0.550	0.423–0.716	**0.001**

Statistically significant *P* values (*P* < 0.05) are in bold. Only values included in the final multivariate model are shown.

HR, hazard ratio; CI, confidence interval; CRT, chemoradiotherapy; RT, radiotherapy.

## Discussion

To the best of our knowledge, this is the largest report assessing practice patterns and outcomes of RT with or without chemotherapy for elderly NPC patients. Our study of a large national database of this relatively uncommon clinical circumstance notably demonstrates that the addition of chemotherapy to RT is independently associated with greater survival in older patients, indicating that the benefit of chemotherapy in NPC may extend potentially to all ages.

A main message from our analysis is that causation is not implied; it could very well be that patients receiving RT alone were not healthy enough to receive additional chemotherapy, and hence they would naturally do worse and be at greater risk of dying from noncancer causes as mentioned above. Although the lack of endpoints such as cancer‐specific survival and local/regional control in the NCDB hampers firm conclusions, there are several reasons to believe this bias may be relatively minimal. First, cohorts were relatively balanced, including no differences in Charlson‐Deyo comorbidity index (although this does not equate to performance status, it did independently predict for OS on Cox multivariate analysis herein). In fact, because groups were overall quite balanced, there was relatively little indication for propensity matching, which would have prohibitively eliminated sample size from an already limited patient population. Furthermore, there were only three variables significantly different between groups on multivariable logistic regression analysis (age, gender, and nodal status); of those three variables, the CRT cohort was younger but had a higher proportion of node‐positive disease and males. Younger age has been shown to associate with more advanced disease 17; node‐positivity and male gender also correlate with poorer prognosis 18, 19, 20. In this manner, consistent with other work, it is plausible that chemotherapy potentially may have been given to a “higher‐risk” population, and that there may be “true” benefits to adding chemotherapy 21, 22.

Despite the large dataset offered by the NCDB, one of its major limitations is a lack of toxicity assessment. To this extent, smaller retrospective reports of older NPC patients (which have employed varying definitions of “older/elderly”) suggest that despite the increase in acute toxicities when adding chemotherapy to RT, these may not be worse in severity from those experienced by younger patients 23, 24. This is consistent with multiple studies in other head/neck neoplasms showing similar toxicities and/or outcomes in elderly patients as compared to their younger counterparts 2, 25, 26, 27. Thus, we encourage judicious and individualized judgment when evaluating administration of CRT in elderly NPC patients; there will likely never be a “definitive answer” regarding aggressive therapies (vs. lack thereof) in elderly patients, owing to retrospective patient selection biases and varying definitions of “older/elderly” patients from study to study.

We, therefore, propose that the term “older/elderly” should not be singularly defined by age, because these patients are intrinsically heterogeneous 28. Rather, utilization of many available measures to measure functionality (e.g., the Comprehensive Geriatric Assessment) and performance status (PS) is a more reliable way to divide “older/elderly” patients into the “functionally older/elderly” or “functionally young” 29. For instance, Liu and colleagues did not find a benefit to adding chemotherapy to RT in NPC patients with high comorbidity indices 23. This study was underpowered to confirm those findings. Nevertheless, these and other parameters are critically important in adequately selecting “elderly” patients that are “fit” to receive aggressive oncologic therapies.

Lastly, although one method to reduce toxicities of CRT is delivering chemotherapy and RT sequentially, we were unable to separately ascertain the benefit of concurrent versus sequential CRT. In our dataset, a large majority (77%) of CRT patients received chemotherapy and RT within 2 weeks of each other (two weeks being a previously utilized cutoff point for concurrent therapy in prior such publications 30). Of the remaining 23% of the CRT cohort, timing of therapies was unknown in 7%, indicating that just 16% certainly received sequential CRT. This was much too small of a sample size to analyze separately in this study. Hence, induction chemotherapy followed by RT remains an attractive option in well‐selected “older” NPC patients at higher risk of toxicities. Additionally, although the use of induction chemotherapy followed by CRT could emerge as a new standard of care 31, the phase III trial deliberately excluded patients ≥60 years of age owing to toxicity risks. However, retrospective data of patients ≥60 years treated with CRT with or without induction chemotherapy showed no outcome differences, with higher toxicities in those receiving induction therapy 32.

Although the NCDB provides a unique platform for studying this important clinical question, this investigation still has limitations. First, NCDB studies are inherently retrospective, with selection biases and lack of several endpoints as mentioned above. Second, NCDB does not keep track of precise chemotherapy details, including specific chemotherapeutic agents, reasons for withholding chemotherapy in RT alone patients (ie. related to tolerability vs. disease‐ related factors), or the number of cycles of chemotherapy received. Third, the NCDB does not allow for an assessment of subsequent lines of treatment (e.g., re‐irradiation, further systemic and/or targeted therapy), which could influence OS. Furthermore, the NCDB also does not provide details such as performance/functional status, Epstein–Barr virus status, or radiotherapy field design/volumes/techniques. Fourth, a major limitation of this study was too few patients for a statistically reliable subset analysis of whether benefit to CRT is limited to those with advanced versus limited nodal disease. The NCDB is also unique to the United States and thus may not be representative to other areas of the world where NPC is endemic.

## Conclusions

This is the largest study to date evaluating the utility of CRT, as compared to RT alone, for older (≥70 years old) patients with NPC. Administration of CRT was independently associated with improved survival, but causation is not implied, and careful patient selection is necessary to balance treatment‐related toxicity risks with potential oncologic benefits.

## Conflict of Interest

None declared. This has never been presented/published before in any form. All authors declare that conflicts of interest do not exist.
